# The International Text-Book of Surgery

**Published:** 1900-03

**Authors:** 


THE INTERNATIONAL TEXT-BOOK OF SURGERY.—By American and British authors. Edited by J. Collins Warren, M. D., LL. D., Professor of Surgery in Harvard Medical School, Boston; and A. Pearce Gould, M. S., F. R. C. S., Lecturer on Surgery and Teacher of Operative Surgery Middlesex Hospital Medical School, London. Vol. I, General and Operative Surgery. With 453 illustrations in the text, and nine full-page plates in colors. 947 pages. Price, $5.00, net. Philadelphia: W. B. Saunders, 925 Walnut Street. 1900.

Another new text-book on surgery will be welcomed by the profession, notwithstanding the many valuable works already published. The advancements in surgery are being made so rapidly that no work can long stand without being thoroughly revised or rewritten altogether. The great volume of new matter coming to the workers in surgery and surgical pathology needs to be utilized, and frequent books are necessary.

The present work will be in two volumes, Volume I having just been issued. It is chiefly devoted to general surgery, while Volume II will deal with the various branches of special surgery.

Volume I is divided into twenty-eight chapters, including surgical bacteriology by Ernst; hyperemia, inflammation, local infection and its termination; suppuration, abscesses, ulcer, sinus, fistula, erysipelas, hospital gangrene, tetanus, operative and plastic surgery, and dislocation of the hip, by Warren; surgical pathology of the blood, by Cabot; Wounds and contusions, burns and scalds, effects of lightning shock; fat embolism, repair of special tissues, by Fowler; constitutional reactions to wounds and their infections, hydrophobia, anthrax, glanders, actinomycosis, madura foot, snake-bite and insect bites, by Van Hook; gangrene, by Spencer; surgical tuberculosis, by Cameron; the technic of aseptic surgery, by McBurney; minor surgery, by DaCosta; anesthetics and surgical anesthesia, by Gay; tumors, by Sutton; fractures, by Pilcher; injuries of the joints and dislocations. by Makins; diseases of the bones, by

Cheyne; diseases of the joints, by Willard; diseases of special joints, by Parker; congenital dislocation of the hip, flat-foot and club-foot, by Bradford; surgery of the muscles, tendons and bursœ, by Monks; criminal surgery, by Tiffany; surgery of spine, by Bird; surgery of the peripheral nerves, by Richardson; surgery of the heart and blood vessels, by Burrell, and surgery of the lymphatic system, by Hamilton.

It is a work that will go into the library of many general practitioners, and will be consulted with much satisfaction. Formerly the general practitioner got along with a single-volume work on surgery, but now he wants the subject in two volumes. The International Text-Book of Surgery is one of the latest and best.

